# Capturing patient experience: does quality-of-life appraisal entail a new class of measurement?

**DOI:** 10.1186/s41687-020-00254-1

**Published:** 2020-10-27

**Authors:** Carolyn E. Schwartz, Roland B. Stark, Bruce D. Rapkin

**Affiliations:** 1 DeltaQuest Foundation, Inc., 31 Mitchell Road, Concord, MA 01742 USA; 2 Departments of Medicine and Orthopaedic Surgery, Tufts University Medical School, Boston, MA USA; 3 Department of Epidemiology and Population Health, Albert Einstein College of Medicine, Bronx, NY USA

**Keywords:** Appraisal, Measurement theory, Clinimetric, Psychometric, Idiometric, Causal indicator, Effect indicator, Formative versus reflective measurement model, Response shift

## Abstract

**Background:**

Two decades of research on quality-of-life (QOL) appraisal have demonstrated links between patient experience and health outcomes and have accounted for both intra-individual change and inter-individual differences in a wide range of research contexts. The present work investigates patterns across diagnostic and demographic groupings to demonstrate how population-specific circumstances drive the structure of QOL appraisal.

**Methods:**

This secondary analysis (*N* = 6448) utilized data from six patient groups: spine surgery, multiple sclerosis, heterogeneous chronically ill, heterogeneous cancer, bladder cancer, and human immunodeficiency virus (HIV). We explored patterns of inter-item correlation across patient samples, using items from the Standards of Comparison and Sampling of Experience subsections of the QOL Appraisal Profile v1 and v2. Similar matrices were compared by demographic characteristics.

**Results:**

Patterns of inter-item correlations for Standards of Comparison items varied sharply across disease groups and racial groups while being similar across age, gender, and education levels. Inter-item correlation matrices for Sampling of Experience items revealed marked differences among disease groups and educational and racial categories but were similar across age and gender groups.

**Conclusions:**

Appraisal parameters showed evidence of shared and unique aspects across samples and circumstances, findings which make sense in light of sample differences in health status and demographic influences. Tools to assess patient experience and meaning may be best understood as *idiometric* instruments. We discuss their distinctions from psychometric and clinimetric tools at theoretical, statistical, and applied levels.

**Supplementary information:**

**Supplementary information** accompanies this paper at 10.1186/s41687-020-00254-1.

## Introduction

Emerging interest from the United States Food and Drug Administration in measuring patient experience [1] points to the importance of understanding quality-of-life (QOL) appraisal. The measurement of appraisal reveals how people recall, prioritize and evaluate experience [2]. Appraisal is especially valuable because of its demonstrated ability to link experience with health outcomes [3, 4]. Further, it enables detection of response-shift effects by operationalizing the ‘contingent true score’ [5]. In other words, the meaning of any QOL score is entirely contingent on individuals’ understanding of measures and their personal criteria for responding to items. Without appraisal assessment, individual differences in understanding obscure the impact on QOL of treatments and of changes in health. By directly assessing QOL appraisal, studies have accounted for both intra-individual change and inter-individual differences in a wide range of research contexts [3, 6–10]. Increasing the personalization of QOL research requires investigation of the individual cognitive processes by which individuals translate their experience into answers on a QOL measure [11].

Efforts to assess cognitive appraisal introduce, however, new and unfamiliar challenges. Appraisal involves description of experience and meta-cognition. These phenomena may be probed at many levels of detail and can be exceptionally fluid. Unlike the evaluation of QOL constructs, there is no particular expectation that appraisal items necessarily converge into internally-consistent dimensions. It is likely that individual cognitive processes differ across individuals with different experiences in sociodemographic or psychosocial factors, diagnosis severity, and illness trajectory [3, 10, 12]. It is also likely that individuals utilize different appraisal processes over time in response to health-state changes (response shift catalysts [2, 13]), and tracking these changes has been identified as a useful way to assess response-shift effects [2, 11, 14–17]. Appraisal assessment has been found to be useful as a way to understand global transition assessments [18] and as a way of linking life goals to psychological outcomes in patients facing terminal illness [19, 20]. It has been suggested as a way to delve deeper into the personal meaning underlying adjustment to acquired disability [21, 22] and to improve the estimation of treatment efficacy in clinical-trials research [23].

In 2004, Rapkin and Schwartz [2] proposed the QOL Appraisal Profile (QOLAP) as a way to capture the cognitive appraisal processes underlying responses to QOL questionnaires. Mirroring separate work by Tourangeau [24], this tool included open- and close-ended items to assess four sets of appraisal parameters: frame of reference, sampling of experience, standards of comparison, and combinatory algorithm. In studies of diverse patient populations, this tool has documented differences in patterns of emphasis between people who fare better, fare worse, or have a variable course with multiple sclerosis (MS) [4], after spinal surgery [8], or before [25] and after invasive cancer treatment [26, 27]. It has documented differences in key indicators of clinically important change, suggesting that interpretation of change over time depends on many factors beyond post-minus-pre scores [28]. Next-generation appraisal measures focused on reducing the need to code text generated from open-ended questions. Statistical modeling enabled us to discern the essential concepts and items of the QOLAP [7], and two close-ended measures were created. The QOLAPv2 contains 85 items, operationalizing the four appraisal parameters separately and relatively comprehensively [12]. The Brief Appraisal Inventory contains 23 items that summarize key patterns found in empirical research using the QOLAP and QOLAPv2 [29], but not distinguishing the underlying appraisal processes. These new measures thus provide practical options for including appraisal measurement in QOL research and clinical settings [30]. Research to date with these measures has documented face-, content-, and ecological validity [31], stability in the context of unchanging health [10], responsiveness in the context of response-shift catalysts [32], and applications as a clinical practice tool [33, 34] and clinical research tool [26]. What we have not observed are consistent correlations among appraisal items from sample to sample.

In these 15 years examining appraisal measures’ inter-item correlations and principal components, we have noted that some themes emerge across samples but that there are also distinct themes in each sample. Given the apparent validity of these appraisal measures in other regards, we do not dismiss this as noise from sample to sample. Rather, this pattern of findings may suggest that there are shared and unique ways that appraisal items co-vary across patient groups, perhaps due to item content, sample composition or human experience. One has to determine in each assessment context whether and how observations hang together and make sense.

These observations have led us to posit that correlations among a given set of cognitive appraisal items need not and should not be the same from population to population. Rather, correlations should be expected to reflect known real-world differences that impact patient experience, as reflected in sample composition and contexts of measurement. In contrast with the psychometric conceptualizations of measurement structure and construct validity which are posited to be universal properties of the measure itself, we believe that appraisal-measure validation involves the behavior of a measure in-context. These individual cognitive processes are thus posited to be non-ergodic, that is they differ across people and change over time (i.e., non-homogeneous across people and non-stationary over time) [35–37]. If so, then a different measurement paradigm should be considered for assessing such phenomena [37]. To date no study has compared different patient groups on the ways that appraisal items relate to each other. The present secondary analysis will thus investigate cross-sectional patterns across diagnostic and demographic groupings. We will then propose a different measurement paradigm applicable to non-ergodic concepts and measures.

## Methods

### Sample and design

This secondary analysis utilized data from six data sets of medically ill populations. These data pulled together different studies with different research questions, all of which shared the use of a common measure. All patients provided written informed consent for use of their data in primary and secondary analyses related to quality of life studies. These data were collected over the past two decades and initially included the QOL Appraisal Profile (QOLAP). Later data collections included the QOL Appraisal Profile-v2. For the present analysis, two appraisal parameters will be considered since they have been included in both the original QOLAP and the QOLAP-v2: Standards of Comparison and Sampling of Experience. Standards of Comparison items (8 rating-scale items ranging from 0 to 6) focused on points of reference considered in evaluating QOL, including for example “Most people your age,” “Your ideal or dream of perfect health,” and “A time in your life before you had this chronic condition.” Sampling of Experience items (14 rating-scale items ranging from 0 to 6) asked individuals about the kinds of situations they recalled in responding about QOL. Items address several theoretical considerations that can enter into memory: valence/mood, primacy/recency, cueing, and the social demand characteristics of the interview. Exemplary items include “Did you find yourself thinking about the worst possible moments,” “Did you try to remember everything relevant over the past three months,” and “Did you try to give your first reaction to the questions.” All of the studies included longitudinal data but for the present analyses only baseline data were used.

Table 1 shows the demographic characteristics of the six samples. Only aggregate information on education was available for the bladder cancer and MS patients, and race information was not available for the MS sample. The total sample of 6448 individuals included 258 spine surgery patients, 859 multiple sclerosis (MS) patients, 2352 chronically ill patients, 1820 heterogeneous cancer patients, 550 bladder cancer patients, and 609 people with human immunodeficiency virus (HIV). The chronically-ill sample included a heterogeneous group of United-States respondents from chronic/rare disease panels comprising patients representing about 350 diagnoses and their caregivers (see www.rarepatientvoice.com for details). While these latter panels included a subgroup of MS patients, their data were collected in a separate study with different measures and design from the MS sample and thus were kept distinct for analysis. Time since diagnosis was available for the MS, RPV, and HIV samples, and the mean years since diagnosis ranged from 7.2 to 15.5 (RPV: 7.2 years; HIV: 9.6 years; MS: 15.5 years). Whereas the spine surgery and bladder cancer patient data were collected via hospital clinics, the HIV data were collected through Medicaid clinics, and the MS, chronically ill and heterogeneous cancer patient data via a patient registry / panel. Supplemental Table 1 provides the descriptive statistics on the appraisal items.

**Table 1:**
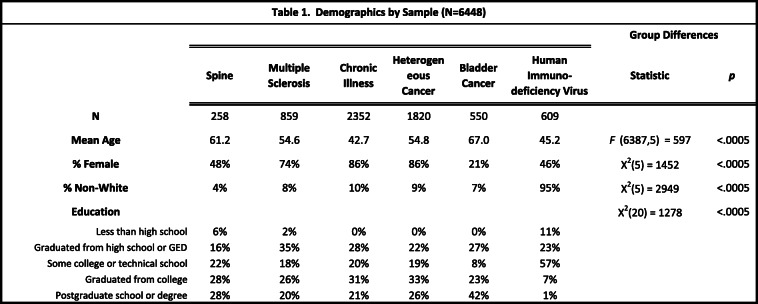
Demographics by Sample (*N*=6448)

### Statistical analysis

We sought to explore patterns of inter-item correlation across patient samples. Separately for the Standards of Comparison and Sampling of Experience item sets, we compared patterns in correlation matrices by patient disease and by demographic group. We used conditional formatting to characterize the direction and magnitude of correlations as per Cohen’s [38] effect size (ES) guidelines: small ES = |0.10–0.29|; medium ES = |0.30–0.49|; large ES = > |0.50|. Positive correlations were rendered in green, and negative in red. The more saturated the color, the larger the ES. This conditional-formatting approach allowed us to visualize patterns across groups, rather than conducting multiple comparisons with over 700 coefficients.

In addition to generating these descriptive results, we conducted inferential tests, separately for the eight Standard of Comparison (SOC) and the 14 Sampling of Experience (SOE) items. These tests examined the extent to which the six patient groups systematically differed in their patterns of inter-item correlations, expressed as distances. To accomplish this, we first transformed each such correlation coefficient (*r*) into its equivalent in the Fisher *Z*_*r*_ metric – a standard transformation to prepare for calculations involving *r* [39]. Next, for a given pairing of the six patient groups, we computed the difference in their corresponding *Z*_*r*_ values (e.g., the difference in the two groups’ *Z*_*r*_ between item 1 and item 2). Since this analysis focused on distances, we took the absolute value of this difference. We then recorded these distances for each item pairing: 8 * 7 / 2 = 28 for the SOC set and 14 * 13 / 2 = 91 for the SOE set. We then repeated these steps for each of the 15 possible patient-group pairings. This yielded a total of 420 SOC distances and 1365 SOE distances. An analysis of variance (ANOVA) for each set allowed a straightforward test of the difference in mean distance according to patient-group pairing. Statistical analyses were implemented using IBM SPSS version 26 [40] and Microsoft Office 365 Excel Version 1902.

## Results

The samples represent a broad range of demographic characteristics, varying by age, proportion female, proportion non-white, and level of education (*p* < 0.0005 for group differences by each variable; Table 1). On the basis of the inter-item correlation matrices, in Standards of Comparison the MS, chronically ill, and heterogeneous cancer samples were most similar to each other. The Spine and Bladder-Cancer were modestly dissimilar from the rest, and the HIV were highly dissimilar (Table 2 displays all of the correlations; Supplemental Table 2 summarizes them by showing the mean *r* within each patient group and within each parameter; Supplemental Table 3 shows correlation matrices with full item content). HIV patient correlations were nearly all distinct from those of other groups. A typical example involves the contrast between the HIV patients (*r* = − 0.04) and the five other groups (0.38 < *r* < 0.55) in the link between the first two items, “Other people you know who are living now with your health condition” and “People whose health doesn’t limit in any way.” As an example in the other direction, the HIV *r* of 0.70 far exceeded those of the other groups (− 0.04 < *r* < 0.49) for the link between “The things your doctor told you would happen?” and “Your ideal; your dream of perfect health?”

**Table 2:**
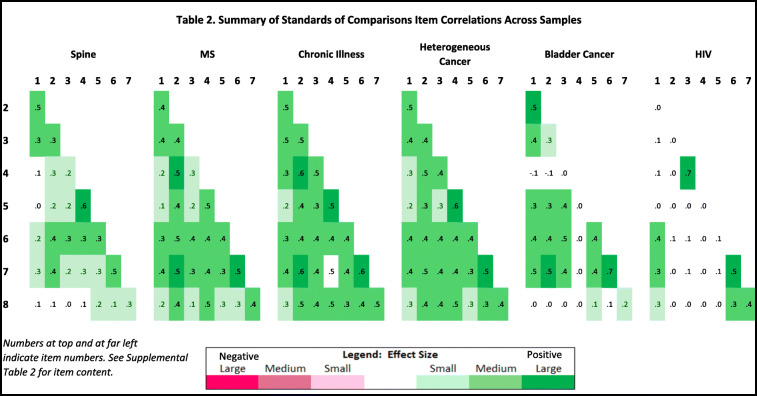
Summary of Standards of Comparisons item correlations across samples

Regarding the Sampling of Experience inter-item correlations, again the spine and bladder-cancer groups were moderately dissimilar from the rest, and the HIV, highly dissimilar (Table 3, Supplementary Table 2, Supplemental Table 4 shows correlation matrices with full item content). For example, for the *r* between the first two items (“Find yourself thinking about the worst moments” and “Emphasize the positive as much as possible”), the value for HIV (− 0.02) contrasts with those for the other groups (− 0.51 < *r* < − 0.17).

**Table 3:**
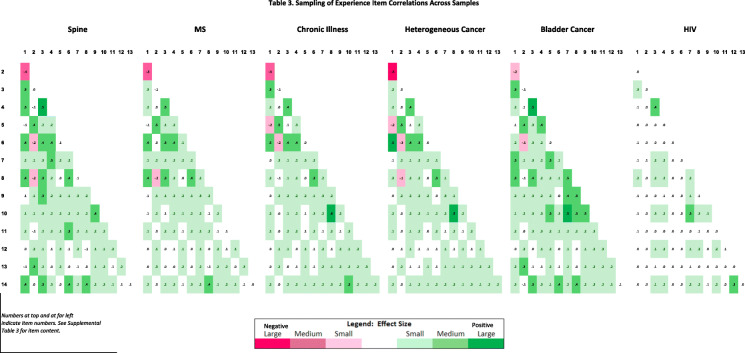
Sampling of Experience item correlations across samples

For Standards of Comparison, analysis by demographic characteristic revealed highly similar inter-item correlations by age group (<=50 versus > 50). For the younger and older patients, the set of correlations involving any given item were highly correlated between groups with an r_range_ = 0.9–1.0. Results were similar by gender (male versus female; r_range_ = 0.9–1.0) and education level (non-college grad versus college grad; r_range_ = 0.9–1.0; Supplemental Table 5). A comparison by racial group revealed somewhat weaker associations (non-white versus white; r_range_ = 0.7–1.0).

For Sampling of Experience, we obtained somewhat lower associations by age group (<=50 versus > 50; r_range_ = 0.7–1.0); and gender (male versus female; r_range_ = 0.6–1.0); and much lower by education level (non-college grad versus college grad; r_range_ = 0.3–1.0) and racial group (non-white versus white; r_range_ = 0.3–1.0; Supplemental Table 6).

**Fig. 1:**
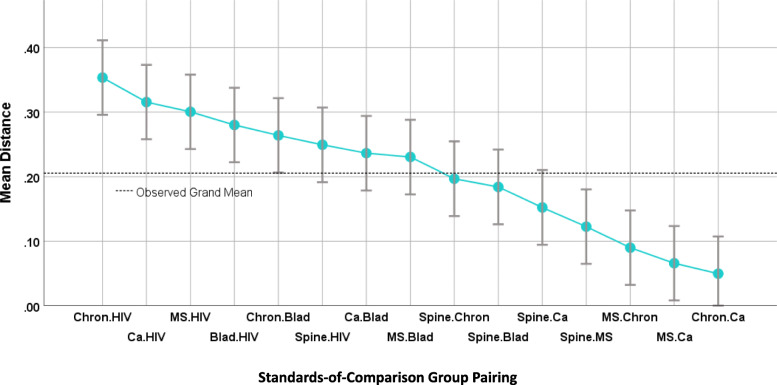
Standards of Comparison: Z-to-r Distance between patient-group pairs for item-to-item correlations. This graph displays results from the first ANOVA. Each of 15 patient-group pairings is characterized on the Y-axis by the mean distance among 28 pairs of item-to-item correlations. Patient-group pairings showing similar item-to-item correlations (low mean distances) appear at right. Mean distances are widely dispersed, ranging from 0.04 to 0.35. 95% confidence-interval error bars are shown above and below the point estimate

**Fig. 2:**
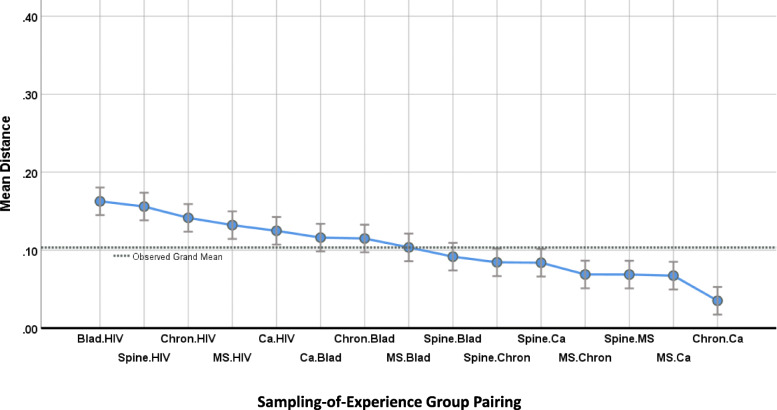
Sampling of Experience: Z-to-r Distance between patient-group pairs for item-to-item correlations. This graph displays results from the second ANOVA. Each of 15 patient-group pairings is characterized on the Y-axis by the mean distance among 91 pairs of item-to-item correlations. Patient-group pairings showing similar item-to-item correlations (low mean distances) appear at right. Mean distances are narrowly dispersed, ranging from 0.04 to 0.16. 95% confidence-interval error bars are shown above and below the point estimate

ANOVA for both the SOC and SOE items sets revealed distinct mean differences in inter-item-correlation distances when analyzed by patient-group pairings. The SOC items’ mean distances by patient-group pairing range from .05 to .35 (*F* (419, 14) = 10.16, *p* < .0001, and adjusted R^2^ = 0.23). In contrast, SOE items’ range only from .04 to .16 (*F* (1364, 14) = 16.23, *p* < .0001, and adjusted R^2^ = 0.14). Figures 1 and 2 show that inter-item correlations involving the HIV group are clearly set apart from those involving other groups. In both plots the distances involving pairings with HIV show the four or five highest means among the 15. At the other end of the scale, both plots show that distances involving heterogenous chronic illness and heterogeneous cancer tend to be low (i.e., inter-item correlations are similar). In both plots the pairings involving these two groups show the two lowest mean distances of the 15, and four out of the lowest five.

## Discussion

We have found that the appraisal items for Standards of Comparison and Sampling of Experience are differentially associated across disease groups. The SOC items’ mean distances tended to be larger than those for the SOE, suggesting that the SOC items are better able than the SOE to systematically distinguish the patient-group pairings. When considered across demographic groups, the differences were less stark, but educational and racial differences remained notable. These findings support the idea that patient groups will differ in the patterns of relationships among the cognitive processes underlying QOL item response. The group differences underscore the importance of circumstances in appraisal response. These circumstantial facets may be useful for more simple applications such as understanding patient satisfaction, as well as for more complex purposes such as detecting response-shift effects [7] via a ‘contingent true score’ [2, 5].

In 2004, we first conceptualized that the measurement of appraisal requires an alternative approach to thinking about psychometrics [5]. At that writing, our focus was on the implications of appraisal for understanding psychometric properties of standard QOL measures. For example, test-retest reliability has to be understood in light of the stability of individual appraisal across measurement occasions. Since then, our experience studying appraisal has led to more clarity regarding the measurement properties of appraisal instruments themselves. We propose that appraisal tools represent a different kind of instrument than is commonly used in QOL research: that appraisal tools are *idiometric* QOL tools, in contrast to psychometric and clinimetric tools.

We choose this new term because we believe that appraisal tools are distinguished from both psychometric and clinimetric tools in three broad and important ways: theoretically; in their implications for statistical analysis; and in their applications in clinical practice and research. We will briefly discuss below each of these dimensions and their empirical support (summarized in Table 4; overlap shown in light grey shading).

**Table 4:**
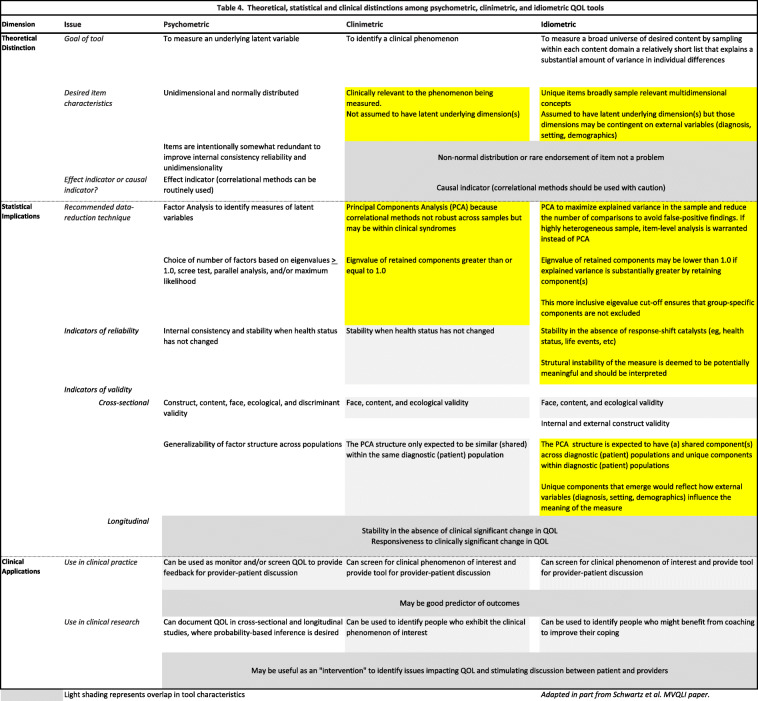
Theoretical, statistical and clinical distinctions among psychometric, clinimetric, and idiometric QOL tools

### Theoretical distinctions among tools

*Psychometric* tools aim to measure a construct comprised of one or more latent variables. Using items that are somewhat redundant within subscales that assess a latent variable, the intention is to achieve a level of internal consistency and unidimensionality that will provide robust results [41]. The items selected are effect indicators (i.e., reflective measurement model [42]), meaning they reflect the latent variable [43, 44] (Supplemental Fig. 1). Relationships among psychometric constructs such as fatigue and depression are essentially understood as being an intrinsic property of those constructs. Such relationships are considered in establishing psychometric construct validity of measures. Ideally, item response covers the full range of response options, and rarely endorsed items are generally dropped early in a tool’s development. The general understanding is that psychometric characteristics such as internal consistency, scale composition, and construct validity are properties of the measure itself and represent the quality of information provided by the measure.

In contrast, *clinimetric* tools (e.g., a measure of symptoms or social/physical environment) aim to identify a (clinical) phenomenon using items that span a broad range of symptoms, so internal consistency and unidimensionality are not priorities [45, 46]. The items selected are sometimes understood as causal indicators (i.e., formative measurement model [42]), meaning that they cause changes in the latent variable of QOL [43, 44] (Supplemental Fig. 1). Rarely-endorsed items are as valuable as commonly-endorsed items because they may help to differentiate clinical syndromes that have overlapping characteristics [45, 46]. Consistent inter-correlations (that is, a similar principal component structure) among items across samples on a clinimetric instrument is not a requirement and indeed, might not even be considered [44]. Rather, quality of clinimetric assessment is more strongly associated with face-validity and construct validity, such as differences among known groups. However, the meaning of clinimetric indicators is expected to be consistent across samples and measurement contexts.

Neither the psychometric nor the clinimetric model quite fits the requirements of appraisal measurement. Appraisal measures are not simply indicators of clinical events or disease-status changes. Appraisal measures are intended to assess the four sets of parameters in the QOL appraisal model. It is reasonable to expect that appraisal processes have both shared (universal) and unique (circumstantial) components that lead to different structures and behaviors across samples and contexts. In contrast to psychometric measures, associations among appraisal constructs may be highly contingent on circumstances. In our experience, appraisal measures do not behave like psychometric measures, but they correlate and explain variance in expected and meaningful ways. Similar to clinimetric tools, such idiometric tools would not emphasize internal consistency or unidimensionality. They would embrace both rarely endorsed and commonly endorsed items.

### Statistical implications of tool differences

Strategies used to validate psychometric tools are not appropriate for use with clinimetric or idiometric measures [43, 44]. Psychometric tools should be able to demonstrate construct validity cross-sectionally in terms of both a factor structure that matches hypothesized constructs and correlations in anticipated directions with other measures of similar and disparate concepts. Such tools should also be able to document content, face, ecological and discriminant validity. The latter three would be the focus for both clinimetric and idiometric measures.

Factor structures of psychometric measures are expected to be consistent across populations, reflecting the generalizability of the constructs. We have not observed such consistency in appraisal parameter structure, but have found that the appraisal measures nevertheless consistently mediate the impact of health status changes on QOL ratings.

This pattern of findings led us to consider the need for an alternative approach to internal and external validity of appraisal measures. We focused on the distinction between construct representation and nomothetic span [41, 47]. Construct representation is demonstrated when item content and form represent the intended constructs (i.e., *internal construct validity*) [41]. In contrast, the nomothetic span refers to a pattern of stronger and weaker relations among measures of the same or different constructs, respectively (i.e., *external construct validity*) [41, 48].

Internal construct validity can be expressed in terms of the observed range of appraisal parameters elicited by a specific measure, relative to the theoretically-specified or expected range [5]. For example, we would expect that self-reported mood state would be more highly correlated with self-reported side effects among those individuals who place greater emphasis on “recent treatment events” in appraising their QOL.

Once we have established an individual’s criteria for appraising QOL, we would need to address external construct validity. (i.e., convergent and discriminant validity) [49]. For appraisal, nomothetic span would mean that the appraisal measure is associated with external constructs in theory-driven ways. Specifically, changes in appraisal might mediate the association between health-status changes (catalysts) and QOL ratings, which is consistent with response-shift theory [2]. A cross-sectional example might be that among individuals who consider “recent treatment events” in appraising their QOL, their ratings would be expected to correlate with a measure of the toxicity of their current treatment regimen [5].

Shared variance among idiometric items is understood as situational rather than intrinsic to the appraisal parameters, so working with item-level data rather than scale scores may be most enlightening. In idiometric analysis as in clinimetrics, variance unique to a single item in the set may be important for understanding patient experiences in different circumstances. Again, scale properties are not assumed to be inherent characteristics of items and measures as in psychometrics, but are instead substantially dependent on contextual influences that can drive inter-item correlations.

If one would prefer to work with sample-specific scale scores, we have found that principal components analysis (PCA) can be an effective data-reduction strategy with appraisal data (e.g., [6]). We have also seen that item correlation patterns can vary markedly across groups. PCA is selected because we do not expect to identify consistent latent factors underlying a set of items that pertain in every situation. We note that PCA may not be the method of choice if the sample is very heterogeneous. For example, one would not analyze in one PCA data from multiple countries with distinct cultures, languages, and healthcare environments. Item-level analyses would be more meaningful in such cases.

Of note, the only overlap in statistical implications for all three types of measures relates to longitudinal validity. Stability and responsiveness are important for all three types of measures. Stability is demonstrated by the lack of change in the absence of a catalyst (e.g., clinically significant change in QOL), and responsiveness is evidenced by the tool’s scores changing when there is a catalytic event.

### Clinical applications of tools

All three types of tools can be used in clinical practice for screening QOL and providing feedback that can facilitate provider-patient communication [33, 34, 50–53], and all may be good predictors of outcomes. While psychometric tools may be used to document QOL, clinimetric and idiometric tools could identify people with a clinical or cognitive characteristic of interest, both of which provide meaningful background to QOL ratings. All tool types may also be used as the basis for an intervention, to identify an individual’s patterns and stimulate helpful discussions with providers.

## Conclusions

In summary, we have proposed and conducted analyses related to considering current QOL appraisal measures to be idiometric tools. We suggest that idiometric measures are distinct at theoretical, statistical, and applied levels from psychometric and clinimetric tools.

Embracing the idiometric concept may spur ideas for improving appraisal measures. Are there, for example, concepts that should be reflected in each parameter that are missing at present? Are there ways to improve how one characterizes the idiometric circumstances for a particular sample, such as defining the stable characteristics of individuals, social determinants of health, and current physical- and mental-health-related contexts? What are the best ways to identify and capture cultural differences in the way a given item is understood? Cognitive-interviewing studies might be helpful to further the validation of these idiometric measures [54].

The present work has clear advantages in the large sample sizes and diversity represented across the six disease groups. Its limitations should, however, be acknowledged. We have used a nomothetic approach, aggregating within patient group and comparing across patient groups. This approach was taken for the purpose of illustrating the importance of circumstances in determining the dimensionality of appraisal measures. We are, however, mindful that within patient groups, there is substantial variation between individuals and within individuals over time. Further exploration of the idiometric nature of appraisal measures would benefit from a deeper look at individual differences in response patterns including for example how time since diagnosis influences appraisal processes. Such deeper looks at individual differences in response patterns should recognize the non-ergodic nature of such constructs in a more person-specific paradigm [37]. We used relatively simple statistical methods to describe patterns and provided a more rigorous test statistic evaluating these differences using ANOVA. In the analysis using inter-item-correlation distances which were analyzed using ANOVA, it is possible that the SOCs' larger inter-item-correlation distances partly stem from the smaller number of distances that make up each pairing’s SOC mean (28 vs. 91). This smaller sample size might well account for some relatively large, random deviations from the mean distance. Nevertheless, given that adjusted R^2^ was 0.23 for SOC versus 0.14 for SOE, we believe that the frequently larger size of SOC distances, and their better ability to systematically distinguish the patient-group pairings, constitutes more than sampling error.

The results are thus illustrative but do not provide a rigorous test statistic. We considered implementing a confirmatory factor analysis but, as noted above, such a latent-variable-based analytic method would not be appropriate for an idiometric measure. The appraisal measures are sampling from a broad ‘universe’ of potential items, rather than reflecting unidimensional latent variables. Formal comparisons of individual bivariate correlations using Fisher’s Z transformation [55, 56] would not address overall pattern differences that we sought to investigate. Future research might develop statistical methods to enable more rigorous testing of the inter-item correlation patterns. A further limitation is that we primarily address validity in the present study. Future work will address indicators of reliability in multiple disease-group comparisons, such as test-retest reliability (stable in the absence of catalysts versus change with catalysts), and internal consistency cross-sectionally and of change scores (in lock step or not).

We hope that the idiometric distinction will lead to a better understanding of ways to work with QOL appraisal data and perhaps of ways to properly characterize other tools that may not behave well using psychometric or clinimetric criteria but still have perceived value. This distinction is consistent with seminal scholarly work calling for an idiographic approach to psychological measurement [35–37]. It is important to validate and analyze measures using methods appropriate to their type.

## Supplementary information

**Additional file 1.**

**Additional file 2: Supplemental Figure 1.** Effect and causal indicators of quality-of-life. Standard psychometric techniques are appropriate for validating effect indicators (i.e., reflective measurement model), which reflect changes in quality of life. In contrast, causal indicators, which cause changes in quality of life (i.e., formative measurement model), would be validated using clinimetric or idiometric methods, depending on the nature of the construct being assessed.

## Data Availability

The data used in these secondary analyses are confidential and thus not able to be shared.

## Abbreviations

ES: Effect size
HIV: Human immunodeficiency virus
MS: Multiple sclerosis
PCA: Principal components analysis
QOL: Quality-of-life
QOLAP: QOL Appraisal Profile
