# Supplementary material for: Capturing patient experience: does quality-of-life appraisal entail a new class of measurement?
Source: J Patient Rep Outcomes. 2020 Oct 27;4:85. doi: 10.1186/s41687-020-00254-1 (PMC7591682; doi:10.1186/s41687-020-00254-1)
Supplement: Supplementary file 1 — Additional file 1. [file 41687_2020_254_MOESM1_ESM.pdf]

Supplemental Table 1. Descriptive Statistics of the Appraisal Items				
	Minimum	Maximum	Mean	Std. Deviation
**Standards of Comparison**				
Others with your condition	0	6	2.36	1.39
Healthy others	0	6	2.74	1.38
Doctor said	0	6	2.41	1.34
Perfect health	0	6	2.9	1.35
Life working for	0	6	3.22	1.26
Way others see you	0	6	2.86	1.31
People your age	0	6	2.86	1.34
Time before health condition	0	6	2.86	1.38
**Sampling of Experience**				
Worst moments	0	6	2.66	1.14
Emphasize positive	0	6	3.47	1.13
Recent few weeks	0	6	3.28	1.11
Relevant past 3 mo.	0	6	3.01	1.17
Balance positive/negative	0	6	3.3	1.17
Recent flare-ups	0	6	2.9	1.29
Future	0	6	3.49	1.26
Focus on health	0	6	3.31	1.27
Relationships	0	6	3.63	1.19
Doctor told	0	6	2.96	1.32
Only for survey	0	6	2.8	1.17
First reaction	0	6	3.63	1.20
Not complain	0	6	3.49	1.25
Seriousness	0	6	3.14	1.28

Supplementary Table 2. Mean Inter-item Correlations by Appraisal Parameter

Patient Sample	*Standards of Comparison (8 items)*						*Sampling of Experience (14 items)*					
	**Spine**	**MS**	**Chronic Illness**	**Cancer**	**Bladder Cancer**	**HIV**	**Spine**	**MS**	**Chronic Illness**	**Cancer**	**Bladder Cancer**	**HIV**
**Spine**	-	0.70	0.64	0.68	0.47	-0.04	-	0.88	0.78	0.82	0.72	0.31
**MS**		-	0.84	0.86	0.22	-0.08		-	0.77	0.80	0.73	0.30
**RPV Chronic Illness**			-	0.90	0.02	0.05			-	0.97	0.60	0.28
**RPV Cancer**				-	0.17	0.06				-	0.64	0.33
**Bladder Ca**					-	0.05					-	0.45
**HIV**						-						-

**Legend: Effect Size**
Large
Medium
Small
Small
Medium
Large

** Conditional formatting shows the magnitude and direction (green = positive; red = negative) of the correlations.*

Supplemental Table 3. Summary of Standards of Comparisons Correlations Across Samples

Spine

	Others with your condition	Healthy others	Doctor said	Perfect health	Life working for	Way others see you	People your age
**Healthy others**	0.49						
**Doctor said**	0.31	0.35					
**Perfect health**	0.10	0.30	0.22				
**Life working for**	0.01	0.23	0.20	0.58			
**Way others see you**	0.21	0.42	0.31	0.35	0.32		
**People your age**	0.27	0.44	0.23	0.26	0.26	0.47	
**Time before health condition**	0.07	0.08	0.03	0.07	0.17	0.10	0.27

MS

	Others with your condition	Healthy others	Doctor said	Perfect health	Life working for	Way others see you	People your age
**Healthy others**	0.38						
**Doctor said**	0.38	0.37					
**Perfect health**	0.17	0.52	0.29				
**Life working for**	0.13	0.36	0.20	0.50			
**Way others see you**	0.30	0.45	0.37	0.37	0.42		
**People your age**	0.35	0.55	0.32	0.42	0.30	0.52	
**Time before health condition**	0.17	0.44	0.15	0.46	0.25	0.29	0.37

Legend: Effect Size

Negative Large	Medium	Small		Small	Medium	Positive Large

Supplemental Table 2. Summary of Standards of Comparisons Correlations Across Samples (continued)

Chronic Illness

Others with your condition	Healthy others	Doctor said	Perfect health	Life working for	Way others see you	People your age
0.48						
0.46	0.47					
0.33	0.56	0.49				
0.24	0.36	0.32	0.54			
0.33	0.43	0.41	0.44	0.44		
0.38	0.56	0.39	0.50	0.39	0.56	
0.28	0.45	0.42	0.48	0.32	0.38	0.50

Heterogeneous Cancer

Others with your condition	Healthy others	Doctor said	Perfect health	Life working for	Way others see you	People your age
0.49						
0.42	0.41					
0.28	0.46	0.40				
0.23	0.33	0.28	0.55			
0.37	0.41	0.38	0.38	0.45		
0.39	0.49	0.36	0.37	0.31	0.52	
0.28	0.45	0.36	0.48	0.30	0.35	0.45

Legend: Effect Size

Negative				Positive		
Large	Medium	Small		Small	Medium	Large

Supplemental Table 2. Summary of Standards of Comparisons Correlations Across Samples (continued)

Bladder Cancer

Others with your condition	Healthy others	Doctor said	Perfect health	Life working for	Way others see you	People your age
0.55						
0.40	0.29					
-0.09	-0.06	-0.04				
0.30	0.31	0.42	0.04			
0.43	0.39	0.45	-0.05	0.45		
0.45	0.51	0.39	-0.04	0.43	0.72	
-0.02	0.02	0.00	-0.02	0.10	0.10	0.16

HIV

Others with your condition	Healthy others	Doctor said	Perfect health	Life working for	Way others see you	People your age
-0.04						
0.06	0.00					
0.06	0.00	0.70				
0.05	0.00	0.00	0.00			
0.35	0.07	0.07	0.05	0.07		
0.34	-0.04	0.06	0.00	0.06	0.52	
0.26	-0.04	0.02	0.05	-0.04	0.34	0.41

Legend: Effect Size

Negative Large	Negative Medium	Negative Small		Positive Small	Positive Medium	Positive Large

Supplemental Table 4. Sampling of Experience Correlations Across Samples

Spine

	Worst moments	Emphasize positive	Recent few weeks	Relevant past 3 mo.	Balance positive/negative	Recent flare-ups	Future	Focus on health	Relationships	Doctor told	Only for survey	First reaction	Not complain
**Emphasize positive**	-0.35												
**Recent few weeks**	0.31	0.01											
**Relevant past 3 mo.**	0.33	-0.07	0.52										
**Balance positive/negative**	-0.08	0.40	0.16	0.22									
**Recent flare-ups**	0.42	-0.20	0.40	0.43	0.07								
**Future**	0.18	0.13	0.29	0.34	0.16	0.17							
**Focus on health**	0.36	-0.16	0.33	0.24	-0.04	0.43	0.10						
**Relationships**	0.09	0.10	0.32	0.24	0.22	0.11	0.30	0.17					
**Doctor told**	0.13	0.15	0.26	0.18	0.20	0.16	0.21	0.22	0.38				
**Only for survey**	0.19	-0.07	0.23	0.25	0.11	0.30	0.16	0.13	0.16	0.17			
**First reaction**	0.05	0.17	0.15	0.09	0.27	0.09	0.19	-0.06	0.14	0.13	0.18		
**Not complain**	-0.05	0.31	0.15	-0.03	0.23	-0.01	0.09	0.02	0.13	0.16	0.09	0.20	
**Seriousness**	0.37	-0.04	0.34	0.29	0.04	0.41	0.22	0.42	0.24	0.25	0.14	0.09	0.06

Legend: Effect Size

Large	Medium	Small		Small	Medium	Large

Supplemental Table 4. Sampling of Experience Correlations Across Samples (continued)

MS

	Worst moments	Emphasize positive	Recent few weeks	Relevant past 3 mo.	Balance positive/negative	Recent flare-ups	Future	Focus on health	Relationships	Doctor told	Only for survey	First reaction	Not complain
**Emphasize positive**	-0.33												
**Recent few weeks**	0.28	-0.08											
**Relevant past 3 mo.**	0.24	0.03	0.48										
**Balance positive/negative**	-0.10	0.48	0.13	0.22									
**Recent flare-ups**	0.37	-0.04	0.34	0.36	0.13								
**Future**	0.13	0.17	0.24	0.24	0.21	0.22							
**Focus on health**	0.41	-0.23	0.31	0.26	-0.03	0.36	0.20						
**Relationships**	0.06	0.14	0.15	0.16	0.17	0.13	0.29	0.21					
**Doctor told**	0.07	0.16	0.10	0.15	0.15	0.18	0.24	0.07	0.24				
**Only for survey**	0.15	0.02	0.15	0.20	0.06	0.17	0.10	0.21	0.05	0.05			
**First reaction**	-0.04	0.14	0.03	0.05	0.14	-0.01	0.11	0.04	0.16	0.09	0.10		
**Not complain**	-0.01	0.29	0.00	0.03	0.22	-0.05	0.17	-0.08	0.16	0.20	0.02	0.26	
**Seriousness**	0.22	-0.02	0.18	0.25	0.10	0.28	0.15	0.38	0.15	0.15	0.12	0.10	0.00

Legend: Effect Size

Large	Medium	Small		Small	Medium	Large

Supplemental Table 4. Sampling of Experience Correlations Across Samples (continued)

Chronic Illness

	Worst moments	Emphasize positive	Recent few weeks	Relevant past 3 mo.	Balance positive/negative	Recent flare-ups	Future	Focus on health	Relationships	Doctor told	Only for survey	First reaction	Not complain
**Emphasize positive**	-0.47												
**Recent few weeks**	0.32	-0.09											
**Relevant past 3 mo.**	0.25	0.01	0.39										
**Balance positive/negative**	-0.23	0.48	0.07	0.28									
**Recent flare-ups**	0.47	-0.21	0.39	0.39	0.03								
**Future**	0.03	0.25	0.14	0.23	0.29	0.17							
**Focus on health**	0.23	-0.06	0.14	0.17	0.03	0.34	0.18						
**Relationships**	0.01	0.16	0.17	0.18	0.21	0.12	0.28	0.19					
**Doctor told**	0.18	0.03	0.12	0.19	0.08	0.27	0.23	0.57	0.23				
**Only for survey**	0.13	-0.01	0.16	0.20	0.07	0.19	0.07	0.19	0.09	0.29			
**First reaction**	0.02	0.10	0.13	0.05	0.10	0.07	0.18	0.09	0.22	0.10	0.16		
**Not complain**	-0.02	0.24	0.04	0.10	0.22	0.07	0.17	0.13	0.17	0.20	0.18	0.28	
**Seriousness**	0.15	0.05	0.15	0.21	0.13	0.21	0.20	0.26	0.23	0.32	0.17	0.17	0.17

Legend: Effect Size

Large	Medium	Small		Small	Medium	Large

Supplemental Table 4. Sampling of Experience Correlations Across Samples (continued)

Heterogeneous Cancer

	Worst moments	Emphasize positive	Recent few weeks	Relevant past 3 mo.	Balance positive/negative	Recent flare-ups	Future	Focus on health	Relationships	Doctor told	Only for survey	First reaction	Not complain
**Emphasize positive**	-0.51												
**Recent few weeks**	0.25	-0.03											
**Relevant past 3 mo.**	0.20	0.04	0.38										
**Balance positive/negative**	-0.20	0.48	0.10	0.26									
**Recent flare-ups**	0.52	-0.26	0.38	0.32	-0.02								
**Future**	0.08	0.21	0.14	0.23	0.23	0.12							
**Focus on health**	0.30	-0.11	0.17	0.17	0.02	0.33	0.13						
**Relationships**	0.02	0.17	0.18	0.19	0.21	0.04	0.28	0.13					
**Doctor told**	0.18	0.03	0.17	0.19	0.11	0.20	0.25	0.51	0.22				
**Only for survey**	0.14	-0.03	0.12	0.17	0.02	0.21	0.06	0.15	0.08	0.19			
**First reaction**	0.02	0.07	0.10	0.09	0.09	0.08	0.11	0.05	0.14	0.10	0.12		
**Not complain**	0.02	0.18	0.10	0.06	0.15	0.06	0.12	0.11	0.11	0.18	0.16	0.24	
**Seriousness**	0.21	-0.02	0.14	0.20	0.11	0.22	0.17	0.29	0.15	0.26	0.11	0.17	0.17

Legend: Effect Size

Large Medium Small Small Medium Large

Supplemental Table 4. Sampling of Experience Correlations Across Samples (continued)

Bladder Cancer

	Worst moments	Emphasize positive	Recent few weeks	Relevant past 3 mo.	Balance positive/negative	Recent flare-ups	Future	Focus on health	Relationships	Doctor told	Only for survey	First reaction	Not complain
**Emphasize positive**	-0.17												
**Recent few weeks**	0.34	-0.08											
**Relevant past 3 mo.**	0.22	0.08	0.52										
**Balance positive/negative**	0.07	0.38	0.25	0.39									
**Recent flare-ups**	0.19	-0.10	0.26	0.22	0.02								
**Future**	0.31	0.12	0.27	0.24	0.32	0.11							
**Focus on health**	0.34	-0.06	0.43	0.23	0.18	0.10	0.45						
**Relationships**	0.24	0.19	0.26	0.15	0.24	-0.02	0.44	0.35					
**Doctor told**	0.11	0.21	0.27	0.25	0.32	0.29	0.54	0.32	0.46				
**Only for survey**	0.24	-0.05	0.25	0.26	0.17	0.12	0.24	0.23	0.18	0.27			
**First reaction**	0.03	0.21	0.01	0.15	0.22	-0.05	0.15	0.02	0.12	0.19	0.27		
**Not complain**	0.17	0.35	0.06	0.07	0.24	0.06	0.19	0.05	0.24	0.18	0.16	0.19	
**Seriousness**	0.29	-0.08	0.34	0.26	0.11	0.38	0.25	0.44	0.21	0.25	0.21	0.12	0.07

Legend: Effect Size

Large	Medium	Small		Small	Medium	Large

Supplemental Table 4. Sampling of Experience Correlations Across Samples (continued)

HIV

	Worst moments	Emphasize positive	Recent few weeks	Relevant past 3 mo.	Balance positive/negative	Recent flare-ups	Future	Focus on health	Relationships	Doctor told	Only for survey	First reaction	Not complain
**Emphasize positive**	-0.02												
**Recent few weeks**	0.28	0.01											
**Relevant past 3 mo.**	0.06	0.03	0.41										
**Balance positive/negative**	0.00	0.00	-0.01	-0.03									
**Recent flare-ups**	0.06	-0.01	0.00	-0.03	0.00								
**Future**	0.07	0.05	0.19	0.16	-0.04	-0.03							
**Focus on health**	0.25	-0.04	0.21	0.23	0.04	-0.04	0.23						
**Relationships**	0.05	0.00	0.03	0.08	0.00	0.00	0.11	0.08					
**Doctor told**	0.10	0.02	0.25	0.21	-0.03	-0.02	0.32	0.29	0.13				
**Only for survey**	0.06	0.00	0.03	0.02	0.00	0.00	-0.03	0.08	0.00	0.02			
**First reaction**	0.01	0.02	0.10	0.24	0.02	-0.03	0.12	0.18	-0.03	0.20	0.09		
**Not complain**	0.03	-0.01	0.08	-0.02	0.00	0.00	0.05	0.00	0.00	0.04	0.00	-0.02	
**Seriousness**	0.01	0.00	0.15	0.22	0.04	0.00	0.20	0.20	0.09	0.21	0.03	0.33	-0.04

Legend: Effect Size

Large	Medium	Small		Small	Medium	Large

Supplemental Table 5. Standards of Comparison Item Correlations by Demographic Groups

	Others with your condition	Healthy others	Doctor said	Perfect health	Life working for	Way others see you	People your age	Time before health condition
**Age <=50 (N ~ 3,000)**								
Others with your condition	.	0.55	0.50	0.27	0.14	0.38	0.42	0.29
Healthy others	0.55	.	0.51	0.48	0.29	0.44	0.56	0.43
Doctor said	0.50	0.51	.	0.48	0.32	0.44	0.47	0.40
Perfect health	0.27	0.48	0.48	.	0.60	0.44	0.48	0.47
Life working for	0.14	0.29	0.32	0.60	.	0.46	0.37	0.32
Way others see you	0.38	0.44	0.44	0.44	0.46	.	0.57	0.40
People your age	0.42	0.56	0.47	0.48	0.37	0.57	.	0.50
Time before health condition	0.29	0.43	0.40	0.47	0.32	0.40	0.50	.
**Age >50 (N ~ 3,000)**								
Others with your condition	.	0.59	0.53	0.33	0.23	0.42	0.47	0.31
Healthy others	0.59	.	0.48	0.49	0.32	0.48	0.54	0.45
Doctor said	0.53	0.48	.	0.47	0.36	0.50	0.47	0.41
Perfect health	0.33	0.49	0.47	.	0.59	0.46	0.47	0.51
Life working for	0.23	0.32	0.36	0.59	.	0.48	0.39	0.37
Way others see you	0.42	0.48	0.50	0.46	0.48	.	0.61	0.42
People your age	0.47	0.54	0.47	0.47	0.39	0.61	.	0.47
Time before health condition	0.31	0.45	0.41	0.51	0.37	0.42	0.47	.
CORRELATION BETWEEN GROUPS	0.99	0.96	0.88	0.98	1.00	0.98	0.91	0.96

Supplemental Table 4 (continued)

	Others with your condition	Healthy others	Doctor said	Perfect health	Life working for	Way others see you	People your age	Time before health condition
**Female (N ~ 4,600)**								
Others with your condition	.	0.55	0.51	0.32	0.22	0.40	0.44	0.31
Healthy others	0.55	.	0.51	0.51	0.34	0.47	0.56	0.46
Doctor said	0.51	0.51	.	0.48	0.35	0.47	0.46	0.42
Perfect health	0.32	0.51	0.48	.	0.58	0.45	0.48	0.49
Life working for	0.22	0.34	0.35	0.58	.	0.48	0.39	0.34
Way others see you	0.40	0.47	0.47	0.45	0.48	.	0.58	0.41
People your age	0.44	0.56	0.46	0.48	0.39	0.58	.	0.50
Time before health condition	0.31	0.46	0.42	0.49	0.34	0.41	0.50	.
**Male (N ~ 1,400)**								
Others with your condition	.	0.63	0.53	0.28	0.13	0.41	0.47	0.29
Healthy others	0.63	.	0.48	0.43	0.27	0.45	0.54	0.38
Doctor said	0.53	0.48	.	0.46	0.34	0.47	0.49	0.38
Perfect health	0.28	0.43	0.46	.	0.63	0.47	0.48	0.48
Life working for	0.13	0.27	0.34	0.63	.	0.46	0.36	0.37
Way others see you	0.41	0.45	0.47	0.47	0.46	.	0.61	0.41
People your age	0.47	0.54	0.49	0.48	0.36	0.61	.	0.46
Time before health condition	0.29	0.38	0.38	0.48	0.37	0.41	0.46	.
CORRELATION BETWEEN GROUPS	0.99	0.90	0.94	0.92	0.98	0.96	0.93	0.90

Supplemental Table 4 (continued)

	Others with your condition	Healthy others	Doctor said	Perfect health	Life working for	Way others see you	People your age	Time before health condition
**Non-White (N ~ 1,000)**								
Others with your condition	.	0.66	0.42	0.17	0.03	0.35	0.39	0.25
Healthy others	0.66	.	0.44	0.31	0.10	0.40	0.44	0.32
Doctor said	0.42	0.44	.	0.42	0.26	0.37	0.43	0.35
Perfect health	0.17	0.31	0.42	.	0.61	0.41	0.41	0.37
Life working for	0.03	0.10	0.26	0.61	.	0.38	0.28	0.28
Way others see you	0.35	0.40	0.37	0.41	0.38	.	0.54	0.38
People your age	0.39	0.44	0.43	0.41	0.28	0.54	.	0.45
Time before health condition	0.25	0.32	0.35	0.37	0.28	0.38	0.45	.
**White (N ~ 4,000)**								
Others with your condition	.	0.50	0.44	0.29	0.18	0.34	0.38	0.25
Healthy others	0.50	.	0.44	0.49	0.32	0.41	0.53	0.42
Doctor said	0.44	0.44	.	0.43	0.29	0.40	0.38	0.38
Perfect health	0.29	0.49	0.43	.	0.55	0.40	0.43	0.47
Life working for	0.18	0.32	0.29	0.55	.	0.44	0.35	0.32
Way others see you	0.34	0.41	0.40	0.40	0.44	.	0.55	0.37
People your age	0.38	0.53	0.38	0.43	0.35	0.55	.	0.46
Time before health condition	0.25	0.42	0.38	0.47	0.32	0.37	0.46	.
CORRELATION BETWEEN GROUPS	0.95	0.72	0.88	0.80	0.94	0.93	0.80	0.80

Supplemental Table 4 (continued)

	Others with your condition	Healthy others	Doctor said	Perfect health	Life working for	Way others see you	People your age	Time before health condition
**Non-College-Grad (N ~ 2400)**								
**Others with your condition**		0.59	0.44	0.20	0.06	0.30	0.37	0.23
**Healthy others**	0.59		0.45	0.39	0.18	0.39	0.50	0.34
**Doctor said**	0.44	0.45		0.41	0.25	0.39	0.42	0.37
**Perfect health**	0.20	0.39	0.41		0.59	0.42	0.43	0.42
**Life working for**	0.06	0.18	0.25	0.59		0.42	0.31	0.31
**Way others see you**	0.30	0.39	0.39	0.42	0.42		0.55	0.37
**People your age**	0.37	0.50	0.42	0.43	0.31	0.55		0.46
**Time before health condition**	0.23	0.34	0.37	0.42	0.31	0.37	0.46	
**College-Grad (N ~ 2300)**								
**Others with your condition**		0.48	0.41	0.28	0.18	0.35	0.36	0.25
**Healthy others**	0.48		0.42	0.47	0.31	0.41	0.51	0.44
**Doctor said**	0.41	0.42		0.42	0.28	0.36	0.33	0.36
**Perfect health**	0.28	0.47	0.42		0.54	0.37	0.40	0.47
**Life working for**	0.18	0.31	0.28	0.54		0.41	0.32	0.30
**Way others see you**	0.35	0.41	0.36	0.37	0.41		0.51	0.34
**People your age**	0.36	0.51	0.33	0.40	0.32	0.51		0.43
**Time before health condition**	0.25	0.44	0.36	0.47	0.30	0.34	0.43	
CORRELATION BETWEEN GROUPS	0.98	0.86	0.85	0.88	0.96	0.88	0.92	0.82

Legend: Effect Size

Negative				Positive		
Large	Medium	Small		Small	Medium	Large

Supplemental Table 6. Sampling of Experience Correlations by Demographic Groups

Age <=50 (N ~ 3,000)	Worst moments	Emphasize positive	Recent few weeks	Relevant past 3 mo.	Balance positive/negative	Recent flare-ups	Future	Focus on health	Relationships	Doctor told	Only for survey	First reaction	Not complain	Seriousness
Worst moments	.	-0.34	0.24	0.18	-0.15	0.49	0.00	0.25	-0.05	0.13	0.22	-0.02	0.00	0.14
Emphasize positive	-0.34	.	0.24	0.23	0.56	-0.06	0.46	0.13	0.45	0.26	0.03	0.36	0.34	0.25
Recent few weeks	0.24	0.24	.	0.51	0.28	0.39	0.41	0.33	0.41	0.32	0.16	0.35	0.19	0.35
Relevant past 3 mo.	0.18	0.23	0.51	.	0.37	0.39	0.36	0.33	0.34	0.32	0.22	0.27	0.19	0.36
Balance positive/negative	-0.15	0.56	0.28	0.37	.	0.12	0.42	0.18	0.39	0.27	0.10	0.32	0.28	0.31
Recent flare-ups	0.49	-0.06	0.39	0.39	0.12	.	0.18	0.35	0.13	0.26	0.24	0.14	0.10	0.29
Future	0.00	0.46	0.41	0.36	0.42	0.18	.	0.36	0.56	0.43	0.10	0.45	0.30	0.41
Focus on health	0.25	0.13	0.33	0.33	0.18	0.35	0.36	.	0.38	0.55	0.24	0.30	0.22	0.40
Relationships	-0.05	0.45	0.41	0.34	0.39	0.13	0.56	0.38	.	0.44	0.12	0.49	0.33	0.41
Doctor told	0.13	0.26	0.32	0.32	0.27	0.26	0.43	0.55	0.44	.	0.25	0.33	0.29	0.42
Only for survey	0.22	0.03	0.16	0.22	0.10	0.24	0.10	0.24	0.12	0.25	.	0.19	0.18	0.19
First reaction	-0.02	0.36	0.35	0.27	0.32	0.14	0.45	0.30	0.49	0.33	0.19	.	0.39	0.41
Not complain	0.00	0.34	0.19	0.19	0.28	0.10	0.30	0.22	0.33	0.29	0.18	0.39	.	0.30
Seriousness	0.14	0.25	0.35	0.36	0.31	0.29	0.41	0.40	0.41	0.42	0.19	0.41	0.30	.
**Age >50 (N ~ 3,000)**														
Worst moments	.	-0.29	0.27	0.29	-0.05	0.55	0.09	0.32	-0.01	0.16	0.29	-0.07	0.03	0.27
Emphasize positive	-0.29	.	0.23	0.20	0.57	`	0.47	0.19	0.43	0.33	0.05	0.43	0.41	0.24
Recent few weeks	0.27	0.23	.	0.50	0.32	0.38	0.37	0.39	0.38	0.35	0.22	0.29	0.25	0.33
Relevant past 3 mo.	0.29	0.20	0.50	.	0.39	0.42	0.37	0.33	0.29	0.32	0.26	0.22	0.20	0.33
Balance positive/negative	-0.05	0.57	0.32	0.39	.	0.14	0.44	0.26	0.40	0.32	0.14	0.35	0.35	0.29
Recent flare-ups	0.55	-0.07	0.38	0.42	0.14	.	0.19	0.36	0.09	0.25	0.33	0.02	0.10	0.32
Future	0.09	0.47	0.37	0.37	0.44	0.19	.	0.42	0.53	0.48	0.13	0.41	0.37	0.37
Focus on health	0.32	0.19	0.39	0.33	0.26	0.36	0.42	.	0.38	0.54	0.26	0.28	0.26	0.47
Relationships	-0.01	0.43	0.38	0.29	0.40	0.09	0.53	0.38	.	0.47	0.10	0.44	0.35	0.33
Doctor told	0.16	0.33	0.35	0.32	0.32	0.25	0.48	0.54	0.47	.	0.23	0.34	0.35	0.40
Only for survey	0.29	0.05	0.22	0.26	0.14	0.33	0.13	0.26	0.10	0.23	.	0.13	0.17	0.24
First reaction	-0.07	0.43	0.29	0.22	0.35	0.02	0.41	0.28	0.44	0.34	0.13	.	0.45	0.30
Not complain	0.03	0.41	0.25	0.20	0.35	0.10	0.37	0.26	0.35	0.35	0.17	0.45	.	0.28
Seriousness	0.27	0.24	0.33	0.33	0.29	0.32	0.37	0.47	0.33	0.40	0.24	0.30	0.28	.
	0.98	0.99	0.92	0.90	0.99	0.96	0.97	0.95	0.98	0.95	0.85	0.94	0.97	0.73
**Female (N ~ 4,600)**														
Worst moments	.	-0.31	0.30	0.29	-0.09	0.54	0.09	0.32	0.03	0.18	0.25	0.02	0.05	0.25
Emphasize positive	-0.31	.	0.19	0.17	0.56	-0.07	0.43	0.10	0.39	0.25	0.05	0.32	0.34	0.22
Recent few weeks	0.30	0.19	.	0.51	0.27	0.41	0.35	0.32	0.36	0.30	0.21	0.29	0.19	0.33
Relevant past 3 mo.	0.29	0.17	0.51	.	0.36	0.43	0.35	0.31	0.30	0.31	0.26	0.22	0.18	0.33
Balance positive/negative	-0.09	0.56	0.27	0.36	.	0.13	0.41	0.18	0.37	0.26	0.12	0.30	0.30	0.27
Recent flare-ups	0.54	-0.07	0.41	0.43	0.13	.	0.22	0.39	0.15	0.28	0.28	0.13	0.13	0.32
Future	0.09	0.43	0.35	0.35	0.41	0.22	.	0.34	0.49	0.41	0.13	0.38	0.30	0.37
Focus on health	0.32	0.10	0.32	0.31	0.18	0.39	0.34	.	0.34	0.54	0.25	0.24	0.20	0.41
Relationships	0.03	0.39	0.36	0.30	0.37	0.15	0.49	0.34	.	0.41	0.13	0.41	0.30	0.35
Doctor told	0.18	0.25	0.30	0.31	0.26	0.28	0.41	0.54	0.41	.	0.25	0.28	0.29	0.39
Only for survey	0.25	0.05	0.21	0.26	0.12	0.28	0.13	0.25	0.13	0.25	.	0.17	0.18	0.21
First reaction	0.02	0.32	0.29	0.22	0.30	0.13	0.38	0.24	0.41	0.28	0.17	.	0.39	0.34
Not complain	0.05	0.34	0.19	0.18	0.30	0.13	0.30	0.20	0.30	0.29	0.18	0.39	.	0.27
Seriousness	0.25	0.22	0.33	0.33	0.27	0.32	0.37	0.41	0.35	0.39	0.21	0.34	0.27	.
**Male (N ~ 1,400)**														
	.	-0.31	0.21	0.15	-0.07	0.52	-0.01	0.21	-0.09	0.06	0.27	-0.11	-0.03	0.14

Age <=50 (N ~ 3,000)	Worst moments	Emphasize positive	Recent few weeks	Relevant past 3 mo.	Balance positive/negative	Recent flare-ups	Future	Focus on health	Relationships	Doctor told	Only for survey	First reaction	Not complain	Seriousness
Worst moments	-0.31	.	0.26	0.27	0.52	-0.10	0.46	0.26	0.46	0.39	0.03	0.46	0.39	0.25
Emphasize positive	0.21	0.26	.	0.48	0.33	0.32	0.46	0.44	0.42	0.40	0.16	0.32	0.26	0.34
Recent few weeks	0.15	0.27	0.48	.	0.40	0.36	0.38	0.35	0.30	0.32	0.20	0.27	0.18	0.38
Relevant past 3 mo.	-0.07	0.52	0.33	0.40	.	0.14	0.43	0.30	0.38	0.35	0.13	0.35	0.31	0.35
Balance positive/negative	0.52	-0.10	0.32	0.36	0.14	.	0.11	0.26	0.03	0.16	0.29	-0.02	0.02	0.27
Recent flare-ups	-0.01	0.46	0.46	0.38	0.43	0.11	.	0.47	0.61	0.54	0.11	0.46	0.37	0.41
Future	0.21	0.26	0.44	0.35	0.30	0.26	0.47	.	0.44	0.54	0.26	0.37	0.32	0.46
Focus on health	-0.09	0.46	0.42	0.30	0.38	0.03	0.61	0.44	.	0.54	0.10	0.48	0.37	0.38
Relationships	0.06	0.39	0.40	0.32	0.35	0.16	0.54	0.54	0.54	.	0.21	0.41	0.36	0.44
Doctor told	0.27	0.03	0.16	0.20	0.13	0.29	0.11	0.26	0.10	0.21	.	0.16	0.17	0.23
Only for survey	-0.11	0.46	0.32	0.27	0.35	-0.02	0.46	0.37	0.48	0.41	0.16	.	0.44	0.36
First reaction	-0.03	0.39	0.26	0.18	0.31	0.02	0.37	0.32	0.37	0.36	0.17	0.44	.	0.30
Not complain	0.14	0.25	0.34	0.38	0.35	0.27	0.41	0.46	0.38	0.44	0.23	0.36	0.30	.
Seriousness														
	0.96	0.96	0.72	0.78	0.96	0.95	0.96	0.62	0.98	0.79	0.94	0.94	0.94	0.85

Non-White (N ~ 1,000)	Worst moments	Emphasize positive	Recent few weeks	Relevant past 3 mo.	Balance positive/negative	Recent flare-ups	Future	Focus on health	Relationships	Doctor told	Only for survey	First reaction	Not complain	Seriousness
Worst moments	.	-0.27	0.14	0.03	-0.15	0.36	-0.13	0.11	-0.11	-0.03	0.19	-0.13	-0.07	-0.11
Emphasize positive	-0.27	.	0.39	0.34	0.53	-0.04	0.54	0.29	0.50	0.47	0.03	0.45	0.36	0.41
Recent few weeks	0.14	0.39	.	0.48	0.37	0.27	0.44	0.35	0.40	0.42	0.10	0.30	0.24	0.35
Relevant past 3 mo.	0.03	0.34	0.48	.	0.38	0.27	0.35	0.35	0.35	0.36	0.18	0.32	0.17	0.33
Balance positive/negative	-0.15	0.53	0.37	0.38	.	0.12	0.45	0.29	0.41	0.39	0.07	0.39	0.30	0.41
Recent flare-ups	0.36	-0.04	0.27	0.27	0.12	.	0.04	0.14	0.06	0.10	0.20	0.04	0.06	0.10
Future	-0.13	0.54	0.44	0.35	0.45	0.04	.	0.44	0.61	0.58	0.06	0.47	0.35	0.49
Focus on health	0.11	0.29	0.35	0.35	0.29	0.14	0.44	.	0.44	0.54	0.24	0.37	0.25	0.40
Relationships	-0.11	0.50	0.40	0.35	0.41	0.06	0.61	0.44	.	0.61	0.13	0.46	0.34	0.47
Doctor told	-0.03	0.47	0.42	0.36	0.39	0.10	0.58	0.54	0.61	.	0.22	0.46	0.32	0.47
Only for survey	0.19	0.03	0.10	0.18	0.07	0.20	0.06	0.24	0.13	0.22	.	0.19	0.16	0.14
First reaction	-0.13	0.45	0.30	0.32	0.39	0.04	0.47	0.37	0.46	0.46	0.19	.	0.44	0.50
Not complain	-0.07	0.36	0.24	0.17	0.30	0.06	0.35	0.25	0.34	0.32	0.16	0.44	.	0.37
Seriousness	-0.11	0.41	0.35	0.33	0.41	0.10	0.49	0.40	0.47	0.47	0.14	0.50	0.37	.

White (N ~ 4,000)	Worst moments	Emphasize positive	Recent few weeks	Relevant past 3 mo.	Balance positive/negative	Recent flare-ups	Future	Focus on health	Relationships	Doctor told	Only for survey	First reaction	Not complain	Seriousness
Worst moments	.	-0.49	0.23	0.21	-0.22	0.49	0.00	0.22	-0.05	0.12	0.14	-0.06	-0.04	0.17
Emphasize positive	-0.49	.	0.06	0.07	0.52	-0.23	0.34	0.02	0.32	0.14	-0.03	0.27	0.30	0.06
Recent few weeks	0.23	0.06	.	0.43	0.17	0.35	0.26	0.24	0.30	0.22	0.13	0.25	0.14	0.21
Relevant past 3 mo.	0.21	0.07	0.43	.	0.31	0.36	0.27	0.20	0.22	0.22	0.17	0.14	0.12	0.23
Balance positive/negative	-0.22	0.52	0.17	0.31	.	0.01	0.34	0.10	0.31	0.17	0.06	0.23	0.25	0.15
Recent flare-ups	0.49	-0.23	0.35	0.36	0.01	.	0.11	0.31	0.05	0.21	0.19	0.03	0.03	0.22
Future	0.00	0.34	0.26	0.27	0.34	0.11	.	0.26	0.44	0.32	0.05	0.33	0.25	0.23
Focus on health	0.22	0.02	0.24	0.20	0.10	0.31	0.26	.	0.27	0.55	0.15	0.17	0.18	0.31
Relationships	-0.05	0.32	0.30	0.22	0.31	0.05	0.44	0.27	.	0.33	0.06	0.37	0.27	0.24
Doctor told	0.12	0.14	0.22	0.22	0.17	0.21	0.32	0.55	0.33	.	0.21	0.21	0.24	0.31
Only for survey	0.14	-0.03	0.13	0.17	0.06	0.19	0.05	0.15	0.06	0.21	.	0.11	0.15	0.13
First reaction	-0.06	0.27	0.25	0.14	0.23	0.03	0.33	0.17	0.37	0.21	0.11	.	0.36	0.22
Not complain	-0.04	0.30	0.14	0.12	0.25	0.03	0.25	0.18	0.27	0.24	0.15	0.36	.	0.19
Seriousness	0.17	0.06	0.21	0.23	0.15	0.22	0.23	0.31	0.24	0.31	0.13	0.22	0.19	.
	0.87	0.91	0.36	0.44	0.91	0.89	0.94	0.49	0.95	0.57	0.86	0.88	0.95	0.27

Non-College-Grad (N ~ 2400)	Worst moments	Emphasize positive	Recent few weeks	Relevant past 3 mo.	Balance positive/negative	Recent flare-ups	Future	Focus on health	Relationships	Doctor told	Only for survey	First reaction	Not complain	Seriousness
Worst moments		-0.43	0.15	0.07	-0.23	0.41	-0.13	0.12	-0.14	0.00	0.17	-0.12	-0.08	-0.02
Emphasize positive	-0.43		0.28	0.24	0.55	-0.12	0.51	0.20	0.49	0.34	-0.02	0.42	0.36	0.33
Recent few weeks	0.15	0.28		0.49	0.32	0.31	0.42	0.34	0.44	0.37	0.12	0.36	0.24	0.36
Relevant past 3 mo.	0.07	0.24	0.49		0.39	0.32	0.37	0.29	0.34	0.31	0.17	0.30	0.19	0.33
Balance positive/negative	-0.23	0.55	0.32	0.39		0.08	0.43	0.22	0.41	0.30	0.06	0.36	0.32	0.34
Recent flare-ups	0.41	-0.12	0.31	0.32	0.08		0.08	0.24	0.08	0.18	0.19	0.09	0.07	0.18

Age <=50 (N ~ 3,000)	Worst moments	Emphasize positive	Recent few weeks	Relevant past 3 mo.	Balance positive/negative	Recent flare-ups	Future	Focus on health	Relationships	Doctor told	Only for survey	First reaction	Not complain	Seriousness
**Future**	-0.13	0.51	0.42	0.37	0.43	0.08		0.40	0.59	0.50	0.04	0.47	0.35	0.45
**Focus on health**	0.12	0.20	0.34	0.29	0.22	0.24	0.40		0.41	0.58	0.17	0.34	0.26	0.38
**Relationships**	-0.14	0.49	0.44	0.34	0.41	0.08	0.59	0.41		0.53	0.07	0.50	0.35	0.46
**Doctor told**	0.00	0.34	0.37	0.31	0.30	0.18	0.50	0.58	0.53		0.19	0.40	0.32	0.44
**Only for survey**	0.17	-0.02	0.12	0.17	0.06	0.19	0.04	0.17	0.07	0.19		0.16	0.14	0.13
**First reaction**	-0.12	0.42	0.36	0.30	0.36	0.09	0.47	0.34	0.50	0.40	0.16		0.44	0.46
**Not complain**	-0.08	0.36	0.24	0.19	0.32	0.07	0.35	0.26	0.35	0.32	0.14	0.44		0.34
**Seriousness**	-0.02	0.33	0.36	0.33	0.34	0.18	0.45	0.38	0.46	0.44	0.13	0.46	0.34	

College-Grad (N ~ 2300)	Worst moments	Emphasize positive	Recent few weeks	Relevant past 3 mo.	Balance positive/negative	Recent flare-ups	Future	Focus on health	Relationships	Doctor told	Only for survey	First reaction	Not complain	Seriousness
**Worst moments**		-0.45	0.25	0.22	-0.20	0.47	0.06	0.25	0.00	0.17	0.12	-0.01	-0.02	0.17
**Emphasize positive**	-0.45		0.00	0.07	0.50	-0.21	0.26	-0.04	0.23	0.09	0.01	0.16	0.25	0.05
**Recent few weeks**	0.25	0.00		0.39	0.09	0.38	0.16	0.17	0.19	0.17	0.12	0.13	0.08	0.15
**Relevant past 3 mo.**	0.22	0.07	0.39		0.27	0.35	0.24	0.19	0.21	0.23	0.16	0.07	0.08	0.21
**Balance positive/negative**	-0.20	0.50	0.09	0.27		0.00	0.28	0.04	0.24	0.15	0.05	0.14	0.16	0.13
**Recent flare-ups**	0.47	-0.21	0.38	0.35	0.00		0.15	0.32	0.08	0.22	0.17	0.02	0.04	0.20
**Future**	0.06	0.26	0.16	0.24	0.28	0.15		0.17	0.34	0.28	0.07	0.18	0.16	0.20
**Focus on health**	0.25	-0.04	0.17	0.19	0.04	0.32	0.17		0.17	0.54	0.17	0.06	0.12	0.29
**Relationships**	0.00	0.23	0.19	0.21	0.24	0.08	0.34	0.17		0.25	0.10	0.20	0.15	0.19
**Doctor told**	0.17	0.09	0.17	0.23	0.15	0.22	0.28	0.54	0.25		0.23	0.11	0.20	0.31
**Only for survey**	0.12	0.01	0.12	0.16	0.05	0.17	0.07	0.17	0.10	0.23		0.10	0.16	0.12
**First reaction**	-0.01	0.16	0.13	0.07	0.14	0.02	0.18	0.06	0.20	0.11	0.10		0.27	0.19
**Not complain**	-0.02	0.25	0.08	0.08	0.16	0.04	0.16	0.12	0.15	0.20	0.16	0.27		0.16
**Seriousness**	0.17	0.05	0.15	0.21	0.13	0.20	0.20	0.29	0.19	0.31	0.12	0.19	0.16	
	0.94	0.93	0.32	0.58	0.92	0.98	0.85	0.56	0.92	0.51	0.88	0.84	0.88	0.32

Legend: Effect Size

Negative						Positive
Large	Medium	Small		Small	Medium	Large
