# Supplementary material for: Capturing patient experience: does quality-of-life appraisal entail a new class of measurement?
Source: J Patient Rep Outcomes. 2020 Oct 27;4:85. doi: 10.1186/s41687-020-00254-1 (PMC7591682; doi:10.1186/s41687-020-00254-1)
Supplement: Supplementary file 2 — Additional file 2: Supplemental Figure 1. Effect and causal indicators of quality-of-life. Standard psychometric techniques are appropriate for validating effect indicators (i.e., reflective measurement model), which reflect changes in quality of life. In contrast, causal indicators, which cause changes in quality of life (i.e., formative measurement model), would be validated using clinimetric or idiometric methods, depending on the nature of the construct being assessed. [file 41687_2020_254_MOESM2_ESM.pdf]

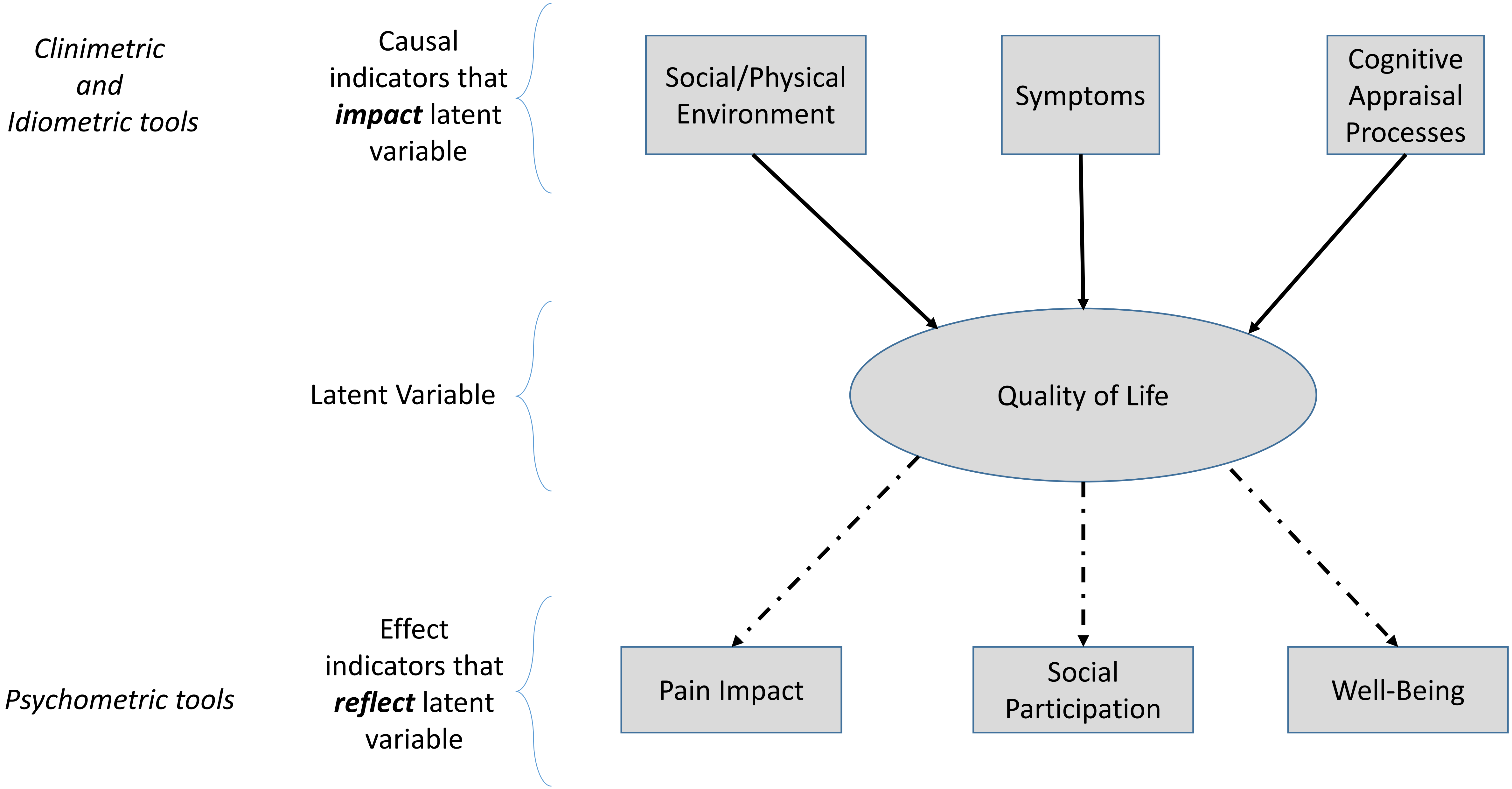
Clinimetric and Idiometric tools
Causal indicators that impact latent variable
Social/Physical Environment
Symptoms
Cognitive Appraisal Processes
Latent Variable
Quality of Life
Psychometric tools
Effect indicators that reflect latent variable
Pain Impact
Social Participation
Well-Being
